# Linking Abusive Supervision to Promotive and Prohibitive Voice Behavior: Testing the Mediating Roles of Work Engagement and Negative Reciprocity

**DOI:** 10.3390/ijerph19095498

**Published:** 2022-05-01

**Authors:** Jialong Wu, Yuechao Du

**Affiliations:** 1 School of Economics & Management, Beijing Forestry University, Beijing 100083, China; jlwu01@bjfu.edu.cn; 2 School of Management, Zhejiang University, Hangzhou 310058, China

**Keywords:** abusive supervision, work engagement, negative reciprocity, promotive voice behavior, prohibitive voice behavior

## Abstract

As an important type of extra-role behavior, employee voice behavior is of great significance to the sustainable development of organizations. Employee voice behavior has two different dimensions, namely promotive voice and prohibitive voice, both of which are conducive to decision making, innovation, and improvements to the work process. Among the antecedents of voice behavior, abusive supervision is one of the most essential influencing factors. In response to the call to further explore the antecedents and influencing mechanisms of different dimensions of voice behaviors, this study aims to investigate the different paths of abusive supervision on the two types of voice behavior. Drawing on the conservation of resources theory and social exchange theory, we identified an expanded array of mediators, including work engagement and negative reciprocity, which link abusive supervision to promotive voice behavior and prohibitive voice behavior separately. Data were collected through two-wave questionnaire surveys of 334 employees of 14 enterprises in China. The results show that (a) abusive supervision is negatively correlated with employees’ promotive and prohibitive voice behaviors; (b) work engagement mediates the negative relationship between abusive supervision and promotive voice; and negative reciprocity mediates the negative relationship between abusive supervision and prohibitive voice. These findings clearly reveal the influencing mechanisms of abusive supervision on both promotive and prohibitive voice behavior, which not only enriches relevant theoretical research but also provides feasible insights into how to reduce abusive supervision to motivate voice behavior in management practice.

## 1. Introduction

COVID-19 has made the business environment increasingly complex and challenging for companies. In the post-epidemic era, companies need to adapt quickly to changing conditions. The development of companies depends not only on the wisdom of leaders but also on the expression of employees’ views and suggestions. Since being presented by Hirschman (1970) in the EVLN model [1], the concept of employee voice behavior has received widespread attention in the field of organizational behavior [2]. Employee voice behavior is considered one of the important manifestations of extra-role behavior [3,4]. This means that employees actively express their opinions or relevant constructive views about work and organizational improvement in the workplace [5,6]. Exploring the antecedents of voice behavior is important not only for improving organizational effectiveness [7] but also for risk avoidance and creating new development opportunities for organizations [8]. Among the many antecedents of voice behavior, leadership behavior has been shown to directly affect employee voice behavior [9]. In prior studies, scholars have mainly discussed the effect of positive leadership on voice behavior; for example, authentic leadership, transformational leadership, and ethical leadership all promote employee voice behavior [10,11,12]. However, as negative leadership behaviors have become more prevalent in recent years, their adverse effects on employees have received continuous attention from scholars. Moreover, negative leadership behaviors have a strong negative effect on organizations and employees, especially employee voice behavior. Therefore, exploring the influence mechanism of negative leadership represented by abusive supervision on employee voice behavior is also of great significance for enriching theoretical research in the field of voice behavior. Recently, some scholars have argued that negative leadership behaviors can inhibit employee voice behavior. For example, studies have shown that when employees are confronted with abusive supervision from their supervisors, they tend to maintain defensive silence [13], correspondingly reducing voice behavior [14,15,16]. Based on the above discussion, an in-depth exploration of the effect of abusive supervision on employee voice behavior is not only valuable in theoretical research but also has a positive impact on management practice.

Abusive supervision is described as the employee’s perception of persistent hostile behavior exhibited by the leader through verbal or nonverbal forms that do not include physical contact [17]. This type of behavior usually includes publicly ridiculing and criticizing subordinates, threatening and intimidating subordinates, making unreasonable demands on subordinates, and appropriating subordinates’ work achievements. Studies have shown that abusive supervision can negatively affect employees’ proactive and responsible behavior [18]. Meanwhile, it also affects employees’ work attitudes, emotional loyalty, and work effort [19,20,21], which further negatively affects extra-role behavior and voice behavior [22,23]. Although existing studies have explored the relationship between abusive supervision and employee voice behavior, most of the above studies were mainly concerned with the effect of abusive supervision on voice behavior as a whole and did not discuss it separately based on the two dimensions of voice behavior. According to Liang Farh, and Farh (2012), employee voice behavior can be divided into two dimensions: promotive voice and prohibitive voice [24]. Promotive voice is an expression of employees’ opinions on improving the efficiency or overall functioning of the organization, while prohibitive voice is defined as preventive suggestions on problems that hinder organizational development [24]. Are the influencing mechanisms by which abusive supervision affects the two types of voice behavior different from each other? Prior studies have pointed out that there are significant differences between the two types of voice behavior. First, the focus and orientation of each type of voice behavior are different. Promotive voice is future-oriented for the organization, and it emphasizes the future benefits and constructive development of the organization. Promotive voice does not have an immediate impact on the organization, but it is closely related to the preservation and acquisition of resources for the organization afterward. Prohibitive voice emphasizes pointing out past and current problems in the organization and focuses more on stopping and reducing losses immediately in the present [24,25]. Second, the risk of engaging in the two types of voice behavior is different. Promotive voice is more easily perceived and accepted by leaders and organizations, which is less risky, while prohibitive voice is mostly regarded as a challenge to the authority of leaders and organizations, and the possibility of successfully practicing prohibitive voice behavior is lower than promotive voice behavior [26,27]. Third, the degree of resource consumption differs between the two types of voice behavior. As a kind of creative behavior, promotive voice is more innovative and constructive. Employees with higher levels of work engagement are usually more creative and are thus able to generate such suggestions [28]. Work engagement is positively correlated with the consumption of cognitive resources. From the motivation to preserve their own resources, when employees are influenced by negative leadership behaviors, they tend to invest less resources, leading to a decrease in work engagement, so that they are not willing to engage in promotive voice behavior, which requires lots of resources [26]. On the other hand, prohibitive voice behavior is more influenced by psychological safety and less influenced by psychological resources. When employees are criticized and distrusted by their leaders or organizations, they generate rebellious and negative emotions. Based on the perspective of social exchange theory, they tend to return bad treatment back to organizations. Thus, influenced by negative reciprocity, when serious problems occur in the organization, employees choose to remain silent to reduce prohibitive voice and make organizations worse [24,27,29]. Therefore, it would be more reasonable to discuss the mechanisms of abusive supervision on each type of voice behavior separately. In general, there are few studies in academia about the effects of abusive supervision on the two types of voice behavior. Additionally, the specific transmission paths and underlying mechanisms are not yet clear.

Currently, scholars usually use psychological ownership theory [30], the role of cognition theory [31], organizational identity theory [32] and other theoretical perspectives to explain employee voice behavior. Voice behavior is usually considered an extra-role behavior with constructive intentions [24,33,34]. However, from the perspective of maintaining personal interests, employees may also use voice behavior as an instrumental means of balancing resources [24,27,35,36]. Based on the conservation of resources theory, when employees suffer from the psychological and work stress caused by abusive supervision [37], they tend to avoid further personal losses to balance their resources. Therefore, they will reduce their work engagement accordingly and will not proactively engage in promotive voice. Meanwhile, unlike promotive voice, which requires more creative ideas and resources to make the organization better in the future, prohibitive voice is less influenced by work engagement and consumes fewer cognitive resources. It is a kind of voice behavior to stop and solve serious problems in the organization at present. It is more likely to be influenced by psychological safety and emotional factors. According to social exchange theory [38,39], when employees are subjected to abusive supervision, they will be affected by negative reciprocity beliefs and will feed this bad treatment back to the organization to varying degrees [40]. When employees are defensive and retaliatory due to negative reciprocity beliefs caused by abusive supervision [40], they will reduce prohibitive voice behavior when the organization has problems. Promotive voice is less affected by negative reciprocity for the following reasons. Negative reciprocity is a sense of revenge that makes employees hope that their organizations get worse. When problems occur in the organization, they choose to remain silent and not address the problems, which can have an immediate and serious negative impact on the organization, leaving it devastated. However, promotive voice is only concerned with the better development of organizations in the future and will not be a critical threat to the organization’s survival and development at present. Without promotive voice, the organizations just remain as usual. Such a kind of voice behavior does not achieve the employees’ purpose of retaliating against the organization. Therefore, to further discuss how abusive supervision influences employee voice behavior, our study uses conservation of resources theory [41] and social exchange theory as a framework to combine work engagement [42,43] and negative reciprocity [44] as separate mediating mechanisms for each of the two types of voice behavior to further explore the relationship between abusive supervision and employee voice behavior.

## 2. Theoretical Background and Hypothesis Development

### 2.1. Abusive Supervision

As a typical representative of destructive leadership, abusive supervision was first introduced by Tepper (2000) and defined as subordinates’ perception of persistent displays of hostile behavior consisting of both verbal and nonverbal forms but not physical contact by leaders [17]. It includes manifestations such as the leader’s public ridicule and criticism of employees, persistent disapproval of employees, and the use of abusive language against employees. According to Tepper (2000), the meaning of abusive supervision can be classified into the following four dimensions: (1) subjectivity of perception; (2) repetition and persistence of behavior; (3) hostile behavior without physical contact; and (4) certainty of behavior occurrence rather than intention. In addition, some scholars have interpreted the concept of abusive supervision from different perspectives. For example, Ashforth (1994) focused more on critiquing leaders for their callousness and abuse of personal authority to mistreat employees [45], while Hornstein (1996) focused more on the purpose of abusive supervision; specifically, leaders will try to control employees by intimidating them [46]. Our study adopts the generally accepted definition of Tepper (2000) for the concept of abusive supervision.

The negative effects of abusive supervision on organizations have become a hot topic as the dark side of leadership behavior continues to draw attention. Regarding the impact of abusive supervision on employees, existing research has focused on several aspects, including employees’ attitudes, mental health, job performance, and workplace behavior. Tepper, Carr, Breaux, Geider, Hu, and Hua (2009) concluded that abusive supervision causes psychological stress, tension, and emotional exhaustion, which affects employees’ psychological well-being [47]. Based on the conservation of resources theory, Harris et al., (2007) proposed that abusive supervision is negatively related to employee performance [48]. Recently, the impact of abusive supervision on employees’ workplace behavior has received increasing attention from scholars. It has been shown that abusive supervision affects employees’ work behavior and leads to work deviance [49,50]. In addition, abusive supervision can negatively affect employees’ other positive behaviors, such as extra-role behavior [18] and knowledge sharing [51], which hinder the sustainability of the organization. In this regard, among the many outcome variables affected by abusive supervision, our study uses voice behavior as the dependent variable to specifically explore how abusive supervision can negatively affect employees’ promotive and prohibitive behavior.

### 2.2. The Relationship between Abusive Supervision and Promotive Voice and Prohibitive Voice

Abusive supervision consists of four dimensions, including subjectivity of perception, repetition, and persistence of behavior, hostile behavior without physical contact, and certainty of behavior occurrence rather than intention [17]. Based on previous studies, we further analyzed and proposed that all four dimensions of abusive supervision had a negative effect on both types of employee voice behaviors. First, when employees perceive the leader’s abusive supervision, they will reduce extra-role behavior. Zellars, Tepper, and Duffy (2002) noted that when employees perceive high levels of abusive supervision, they lose trust in their supervisors and balance their perceived unfairness by reducing extra-role behavior [18]. This perception varies from person to person. Employees with different characteristics perceive the same level of abusive supervision differently and thus behave in a different way [52]. Second, persistent abusive supervision can affect employees’ psychological safety [10]. Employee voice behavior, as an extra-role behavior, is of high risk. If abusive supervision is only occasional, the impact on employee voice is likely to be low. However, when leaders practice persistent abusive supervision of employees, employees will judge the probability of success of their suggestions to be low, which may bring about a negative evaluation of themselves, thus giving up their suggestions and remaining silent [9].

In addition, since Liang, Farh and Farh (2012) divided employee voice behavior into two dimensions, promotive and prohibitive voice [24], studies discussing negative leadership behavior separately for both types of voice behaviors have also emerged. For example, Chamberlin, Newton, and Lepine (2016) argued that as a positive voice behavior, promotive voice can be negatively influenced by negative leadership behavior [53]. Li, Liang, and Liu (2009) argued that employees’ prohibitive voice is moderated by organizational support [54]. Abusive supervision makes employees perceive less organizational support and thus reduces prohibitive voice. Based on conservation of resources theory, employees subjected to abusive supervision would refrain from engaging in promotive voice to conserve resources, as promotive voice behavior would cost their resources a lot. Based on social exchange theory, employees who are treated with hostility by their leaders are highly likely to feed this negative sentiment back to the organization, hiding their suggestions on issues that hinder the development of the organization and thus reducing prohibitive voice. Therefore, based on the above analysis, this study proposes the following hypothesis:

**Hypothesis** **1a** **(H1a).** *Abusive supervision is negatively correlated with employee promotive voice behavior.*

**Hypothesis** **2a** **(H2a).** *Abusive supervision is negatively correlated with employee prohibitive voice behavior.*

### 2.3. The Mediating Role of Work Engagement between Abusive Supervision and Promotive Voice

Work engagement refers to an employee’s ability to be fully engaged in the work role in a multifaceted way. Kahn (1990) first identified physical, cognitive, and emotional dimensions of work engagement [42]. Saks (2006) showed that work characteristics and organizational support can promote work engagement [55]. When employees perceive abusive supervision from their leaders, they may not feel supported by the organization. They probably experience a shift in their attitudes toward proactive engagement in tasks, which in turn may affect their work engagement. Meanwhile, when employees are subjected to abusive supervision, they will experience stress, tension, and psychological depression, which affect their emotional well-being and happiness [17,47,56]. Specifically, employees’ psychological climate, such as work attitudes, positive emotions, perceptions of their job role and self-expression, can be transformed, thus negatively affecting work engagement [57,58]. Based on the above analysis, this study proposes the following hypothesis.

**Hypothesis** **1b** **(H1b).** *Abusive supervision is negatively correlated with work engagement.*

Liang, Farh and Farh (2012) classified employee voice behavior into two dimensions: promotive and prohibitive voice [24]. Kataria, Garg, and Rastogi (2013) concluded that higher work engagement positively predicts employees’ extra-role behavior [57]. Additionally, as a kind of creative behavior, promotive voice is innovative and constructive. Many studies have also shown that employees with higher work engagement are usually more creative and are thus able to engage in such behavior [26,59]. Therefore, it can be inferred that when lower work engagement is generated, employees may reduce some of their extra-role behaviors to maintain their sense of self-control, for example, decreasing their promotive voice behavior that contributes to the efficient functioning of the organization. Moreover, our inference in this study is also consistent with the relevant research on employee stress based on the conservation of resources theory. The theory suggests that when employees become stressed, they may act in this way to eliminate psychological tension and frustration: stop consuming resources immediately to preserve existing resources [41,60]. Additionally, since work engagement is positively correlated with the consumption of cognitive resources, a decrease in work engagement can help employees reduce resource consumption and preserve available resources. Therefore, under the influence of negative leadership behaviors, employees with much stress will stop losing time to preserve their resources. When employees are less engaged in their work, they tend to engage less in behaviors that require many resources [29]. Accordingly, we argue that when employees perceive abusive supervision and generate work stress [37], to preserve their available resources, they may correspondingly reduce their work engagement. Thus, they will not take the initiative to engage in promotive voice conducive to organizational efficiency and long-term sustainability. From the above analysis, it is clear that abusive supervision has a negative impact on employees’ work engagement, which in turn affects employees’ promotive voice behavior, and work engagement mediates the relationship between abusive supervision and employee promotive voice behavior. Therefore, this study proposes the following hypothesis:

**Hypothesis** **1c** **(H1c).** *Work engagement mediates the negative relationship between abusive supervision and employee promotive voice behavior.*

### 2.4. The Mediating Role of Negative Reciprocity between Abusive Supervision and Prohibitive Voice

Reciprocity is a cultural phenomenon that has been prevalent in the development of human society. Gouldner (1960) defined reciprocity as a norm in which one party is obliged to reciprocate when the other party provides help [38]. Later, based on the work of Liden, Sparrow, and Wayne (1997), Uhl-Bien and Maslyn (1997) divided the forms of reciprocity into negative and positive reciprocity [44,61]. According to social exchange theory, negative reciprocity refers to the behavior in which when individuals are treated unkindly by others, they will develop a retaliatory psychological state of not wanting others to be better, thus giving the same bad treatment back to those who are unkindly [39,40]. Mitchell and Ambrose (2007) concluded that abusive supervision makes employees more likely to engage in abnormal behavior that is detrimental to the development of the organization. Employees with a high level of negative reciprocity believe that their supervisors will increase abusive supervision of them [62]. Therefore, it can be hypothesized that abusive supervision is positively related to negative reciprocity. In other words, when employees perceive stronger hostile treatment from their supervisors, with greater negative emotions generating, their antipathy toward their supervisors and organization is stronger; thus, the effect of negative reciprocity is stronger. Accordingly, this study proposes the following hypothesis:

**Hypothesis** **2b** **(H2b).** *Abusive supervision is positively correlated with negative reciprocity.*

**Figure 1:**
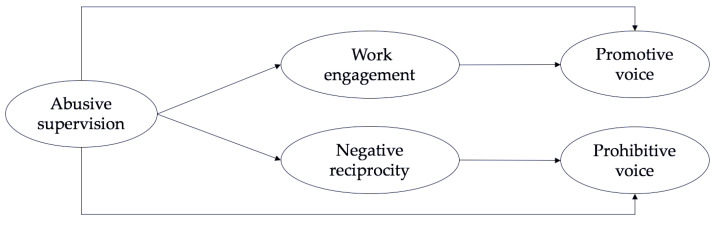
The proposed model.

As another dimension of employee voice behavior, prohibitive voice is a preventative way for employees to express their views on issues that hinder the development of the organization. According to social exchange theory, when employees are treated with hostility, they become defensive and vindictive, feeding back negative feelings to their leaders and organization and not wanting the organization to get better. Therefore, they do not make efforts or contributions to solve problems arising in the organization. Uhl-Bien and Maslyn (2003) suggested that negative reciprocity can lead to negative moods and can also reduce the degree of psychological safety and organizational identification [44]. When employees’ psychological safety and organizational identification decrease, their tendencies to retaliate caused by negative reciprocity will increase; thus, they tend to return bad treatment back to their organizations and hope for major organizational failures. As a result, employees will choose to remain silent and reduce prohibitive voice to retaliate against the organization when there are serious problems in the organization [2,40,44,62]. Therefore, it can be inferred that due to the influence of negative reciprocity beliefs, employees who are subjected to abusive supervision will decrease the prohibitive voice behaviors that maintain the organization’s development when problems are emerging. In other words, negative reciprocity inhibits the generation of an prohibitive voice. Based on the above analysis, the following hypothesis is proposed in this study. Figure 1 shows the proposed model.

**Hypothesis** **2c** **(H2c).** *Negative reciprocity mediates the negative relationship between abusive supervision and prohibitive voice*.

## 3. Method

### 3.1. Sample and Procedure

The data were collected through two-wave surveys from 14 companies in eastern China. First, two companies in Shandong Province were selected for the pre-survey. Based on the suggestions of the respondents, the questionnaire was adapted and modified. A formal survey was conducted with 384 employees from Shandong, Jiangsu, Zhejiang, and Fujian Provinces. Of the 14 companies surveyed, we found that all had previously perceived abusive supervision. The survey was conducted in two stages. In the first stage (Time 1), we measured and collected employees’ demographic information, their work engagement, negative reciprocity, and their perceived level of abusive supervision. In the second stage, four weeks later (Time 2), employees evaluate their promotive and prohibitive voice behavior. Ultimately, the questionnaire was completed and validated for 334 employees, with a valid response rate of 86.98%. Demographically, 173 respondents were male (51.80%). The average age of employees was 40.44 years (SD = 9.45). In terms of education, 255 respondents had junior college or bachelor’s degrees (76.35%). The average organizational tenure of the respondents was 4.37 years (SD = 2.17).

The original scales used in this paper were drawn from previous empirical studies. First, the scales were translated using the translation-back-translation procedure [63]. A few modifications were made to the scale to suit the Chinese cultural context. Second, all variables were scored using a 5-point Likert scale. A higher score means that the respondent is more likely to have the situation and behavior corresponding to the question item. For the remaining variables, the options represent the degree of conformity, ranging from 1 to 5, corresponding to “not at all” to “completely”.

### 3.2. Measures

Abusive supervision. This study used a reduced 10-item version of the initial scale developed by Aryee, Chen, Sun, and Debrah [20], with representative questions such as “My leader makes negative comments about me to others” and “My leader makes fun of me”. The Cronbach’s alpha for this measure was 0.91.

Work engagement. This study uses a 9-item version of the scale developed by Schaufeli, Bakker, and Salanova [43], which is divided into three dimensions, vigor, dedication, and absorption, with representative questions such as “I am immersed in my work”, “I feel strong and energetic when I work” and “I am proud of the work I do”. The Cronbach’s alpha for this measure was 0.95.

Negative reciprocity. This study uses a 14-item version of the scale developed by Eisenberger et al. [40], with representative questions such as “If someone dislikes me, I dislike them too” and “When someone hurts me, I retaliate in an unexpected way”. The Cronbach’s alpha for this measure was 0.94.

Employee voice behavior. This study uses a 10-item version of the scale developed by Liang, Farh and Farh [24], divided into promotive voice and prohibitive voice dimensions, with representative questions such as “I will actively propose new solutions that will benefit the company” and “I will promptly discourage other employees in the company from misbehaving in a way that affects productivity”. The Cronbach’s alpha for this measure was 0.95.

Control variables. According to previous research, demographic variables also influence employee voice behavior to some extent [10,64]. Therefore, in this paper, gender, age, education, and organizational tenure were selected as control variables and categorized in a continuous coding approach. Gender was coded as 0 for female and 1 for male; education was coded as 1 for less than high school, 2 for high school, 3 for junior college, 4 for bachelor’s degree, and 5 for postgraduate and above.

## 4. Results

### 4.1. Common Method Bias Test

Given the impact of the self-report questionnaire and the homogeneity of the data sources, there are issues of common method bias. Therefore, this study adopted Harman’s one-way test and used SPSS26 to conduct an exploratory factor analysis of all scale items, and the first factor could explain only 28.95% of the variance, indicating that common method bias is not a serious problem in our study.

### 4.2. Descriptive Statistics

The descriptive statistics and correlation coefficient matrices for the variables are detailed in Table 1. From Table 1, it can be seen that abusive supervision and work engagement were significantly negatively correlated (*r* = −0.22, *p* < 0.01), and work engagement was significantly positively correlated with promotive voice (*r* = 0.53, *p* < 0.01). Abusive supervision and negative reciprocity were significantly positively correlated (*r* = 0.15, *p* < 0.01), negative reciprocity was significantly negatively correlated with prohibitive voice (*r* = −0.15, *p* < 0.01), and abusive supervision was significantly negatively correlated with both promotive and prohibitive voice (*r* = −0.27, *p* < 0.01; *r* = −0.20, *p* < 0.01). We also calculated the variance inflation factors (VIFs) to test for multicollinearity. The VIFs for all variables were below 5 (1.09 for abusive supervision and 1.07 for work engagement; 1.05 for abusive supervision and 1.07 for negative reciprocity), indicating that there was no serious problem of multicollinearity in this study [65]. The above results are consistent with the research hypothesis of this paper.

### 4.3. Confirmatory Factor Analysis

We used Mplus8 to conduct confirmatory factor analysis to verify the validity of the key variables in our model (displayed in Table 3). This study discusses the convergent and discriminant validity of constructs by comparing the five-factor model, the four-factor model, the three-factor model, the two-factor model, and the one-factor model. The results show that the five-factor model fits best in relative terms. (χ^2^/df = 2.416, CFI = 0.895, TLI = 0.889, RMSEA = 0.065, and SRMR = 0.054). The observable data had a significant loading on the expected underlying factors. To further test the reliability of our model, the hypothetical five-factor model was compared with four other alternative models. The first is a four-factor model that combines promotional voice and prohibitive voice into one latent factor. (χ^2^/df = 2.514, CFI = 0.887, TLI = 0.881, RMSEA = 0.067, and SRMR = 0.055). The second was to combine work engagement and negative reciprocity into one latent factor, with employee voice behavior and abusive supervision as a three-factor model (χ^2^/df = 5.382, CFI = 0.673, TLI = 0.655, RMSEA = 0.115, and SRMR = 0.174). The third is a two-factor model with employee voice behavior, work engagement, and negative reciprocity load on one latent factor (χ^2^/df = 7.572, CFI = 0.508, TLI = 0.483, RMSEA = 0.14, and SRMR = 0.178). The fourth is a two-factor model in which abusive supervision, work engagement, and negative reciprocity were loaded on one latent factor (χ^2^/df = 7.42, CFI = 0.519, TLI = 0.495, RMSEA = 0.139, and SRMR = 0.188). The fifth is a one-factor model in which all the factors were loaded on a single factor (χ^2^/df = 9.292, CFI = 0.379, TLI = 0.348, RMSEA = 0.158, and SRMR = 0.201). A comparative analysis of the data in Table 2 shows that our hypothetical five-factor model fits significantly better than the other models, indicating that the measurement scales have better convergent and discriminant validity.

### 4.4. Hypothesis Testing

The hypotheses of this study were tested through hierarchical multiple regression. As shown in Table 3, Model 1 regressed the effect of control variables on work engagement. Model 2 regressed the effects of abusive supervision and control variables on work engagement. Model 3 regressed the effect of control variables on negative reciprocity. Model 4 regressed the effects of abusive supervision and control variables on negative reciprocity. Model 5 regressed the effect of control variables on promotive voice. Model 6 regressed the effects of abusive supervision and control variables on promotive voice simultaneously. Model 7 regressed the effect of abusive supervision, work engagement, and control variables on promotive voice. Model 8 regressed the effect of control variables on prohibitive voice. Model 9 regressed the effect of abusive supervision and control variables on prohibitive voice. Model 10 regressed the effect of abusive supervision, negative reciprocity, and control variables on prohibitive voice.

**Table 3:** Results of hierarchical multiple regression.

	WE		NR		Promotive Voice			Prohibitive Voice		
	M1	M2	M3	M4	M5	M6	M7	M8	M9	M10
CV										
Gender	0.01	−0.03	−0.07	−0.04	0.16	0.12	0.12	0.14	0.12	0.1
Age	−0.007	−0.009	−0.003	−0.002	0.006	0.004	0.008	−0.002	−0.003	−0.004
Education	0.1	0.08	−0.08	−0.07	0.09	0.07	0.03	0.05	0.03	0.02
OT	0.05	0.04	−0.07 **	−0.07 **	0.04	0.04	0.02	0.03	0.03	0.02
IV										
AS		−0.23 **		0.13 **		−0.28 **	−0.16 **		−0.20 **	−0.19 **
Mediator										
WE							0.51 **			
NR										−0.11 *
R^2^	0.02	0.06	0.05	0.07	0.04	0.1	0.32	0.02	0.05	0.06
F	1.64	4.35 **	4.61 **	4.71 **	3.38 *	7.08 **	26.23 **	1.32	3.33 **	3.48 **

Notes: * *p* < 0.05, ** *p* < 0.01. CV = control variable; IV = independent variable; WE = work engagement; NR = negative reciprocity; OT = organizational tenure; AS = abusive supervision.

According to hypotheses H1a and H2a of this study, abusive supervision is negatively related to both types of employee voice behavior. To test this hypothesis, employee voice behavior was first set as the dependent variable, followed by the addition of control variables (age, gender, education, and organizational tenure) and finally the independent variable, abusive supervision. Model 6 regressed the effect of abusive supervision and control variables on promotive voice behavior, and Model 9 regressed the effect of abusive supervision and control variables on prohibitive voice behavior. As seen in Table 3, abusive supervision has a significant negative effect on both promotive voice and prohibitive voice (Model 6 and Model 9; *b* = −0.28, *p* < 0.01; *b* = −0.20, *p* < 0.01). Therefore, hypotheses H1a and H2a were proven. According to Baron and Kenny (1986), for a mediation effect to be valid, the following conditions must be fulfilled: (1) the independent variable is significantly correlated with the dependent variable; (2) the independent variable is significantly correlated with the mediating variable; (3) the mediating variable is significantly correlated with the dependent variable; and (4) controlling for the independent variable, the mediator explains the dependent variable significantly, and the dependent variable is less correlated with the independent variable, which indicates a partial mediation effect [66]. Therefore, in Models 1 and 3, we regressed the control variables on work engagement and negative reciprocity, respectively. From Model 2 and Model 4 in Table 3, it is clear that there is a significant negative effect of abusive supervision on employees’ work engagement (*b* = −0.23, *p* < 0.01). In contrast, abusive supervision has a significant positive effect on employees’ negative reciprocity (*b* = 0.13, *p* < 0.01). Hypotheses H1b and H2b were proven by the data. In Model 7, we regressed control variables, independent variables, and work engagement on promotive voice, and the results show that work engagement is significantly positively related to promotive voice behavior (*b* = 0.51, *p* < 0.01). In Model 10, we regressed control variables, independent variables, and negative reciprocity on prohibitive voice behavior, and the results show that negative reciprocity is negatively related to prohibitive voice (*b* = −0.11, *p* < 0.05). Therefore, hypotheses H1c and H2c are supported. 

Table 4 and Table 5 show the results of the mediating effects for both pathways. Abusive supervision is significantly negatively correlated with both promotive voice (*b* = −0.28, *p* < 0.01) and prohibitive voice (*b* = −0.20, *p* < 0.01). Excluding the indirect effects of mediating influences, the direct effects of abusive supervision on both voice behaviors are displayed in Table 4 and Table 5 (*b* = −0.16, *p* < 0.01; *b* = −0.19, *p* < 0.01). We also used the bootstrapping method to verify the indirect impacts of mediating effects [67,68]. The indirect effect of abusive supervision on promotive voice behavior via work engagement is −0.12 (*p* < 0.01), and the indirect effect of abusive supervision on prohibitive voice behavior via negative reciprocity is -0.01 (*p* < 0.01). According to MacKinnon and Dwyer (1995), the indirect effect of abusive supervision on promotive and prohibitive voice behavior is not zero, so the mediating effect can be verified [69].

## 5. Discussion

Based on Liang, Farh and Farh (2012), this study subdivides employee voice behavior into two dimensions: promotive voice and prohibitive voice [24]. In view of the different connotations of the two types of employee voice behaviors and their various contributions to organizational development, this study examines the influencing mechanisms of abusive supervision on promotive voice and prohibitive voice from the perspectives of conservation of resources theory and social exchange theory, respectively. Therefore, this study not only has some theoretical contributions but also provides a good reference for management practice.

### 5.1. Theoretical Implications

First, this study broadens the research scope of abusive supervision by revealing the negative direct and indirect impacts of abusive supervision on employee voice behavior. Although most studies have suggested that abusive supervision negatively affects employee voice behavior [70], there are also studies that suggest other correlations, such as an inverted U-shaped relationship [71]. Therefore, based on the contradictions in previous research, this study further explored and ultimately found that abusive supervision negatively affects both types of employee voice behaviors.

Second, based on Liang, Farh and Farh’s study, we divided employee voice behavior into two dimensions to explore the specific influencing mechanisms of abusive supervision on each of them. According to Liang, Farh and Farh (2012), promotive voice is an expression of employees’ opinions on improving the efficiency or overall functioning of the organization; prohibitive voice is when employees anticipate or see problems in the organization’s development and make suggestions on problems that hinder organizational development [24]. Different factors in the organization can have different effects on the two types of employee voice behavior. Therefore, this study explores the impact of abusive supervision on employee voice behavior in two dimensions and explains the influencing mechanisms. When employees face stress and negative emotions from abusive supervision by their leaders, they are less engaged and less focused on conserving resources. Thus, they do not take the initiative to engage in promotive voice behavior. Meanwhile, employees may also return negative emotions to the organization as a result of the unfriendly behavior displayed by supervisors, and they may not make preventive suggestions when organizational problems emerge.

Third, based on the conservation of resources theory, we introduced work engagement as a mediating variable to explain why abusive supervision negatively affects employees’ promotive voice, expanding the application of conservation of resources theory in the relationship between abusive supervision and promotive voice. Abusive supervision can cause employees to experience negative emotions and work stress toward the organization [37]. To safeguard personal interests and preserve available resources, employees may reduce their work engagement and therefore decrease promotive voice behavior, which contributes to the sustainable development of the organization.

Finally, in the view of social exchange theory, we introduced the concept of negative reciprocity as a mediating variable between abusive supervision and prohibitive voice, extending the application of negative reciprocity. Influenced by negative reciprocity, employees become defensive and vindictive in the face of negative leadership behavior [40], thus reducing their prohibitive voice toward the organization. This means that the stronger the degree of abusive supervision perceived by employees, the stronger the effect of their negative reciprocity, and the more inclined they are to remain silent and give up their suggestions when problems arise in the organization.

### 5.2. Practical Implications

In the post-epidemic era, leaders, organizations, and employees are faced with a variety of challenges. How to resolve the impact of supervisors’ abusive supervision on employee voice behavior and how to promote better and faster organizational development are all worthy of consideration. This study provides a reference for this issue in the following three areas.

First, regardless of the type of employee voice behavior, abusive supervision can reduce employee voice behavior. Therefore, supervisors should communicate positively and effectively with employees to understand their own thoughts on behaviors. At the same time, some rules or criteria for rewards and punishments as well as relevant monitoring mechanisms can be established at the organizational level to control leaders’ behavior of abusive supervision [52] so that they can find better management methods to reduce or avoid abusive supervision behavior.

Second, abusive supervision is a subjective perception of employees. Strengthening and developing employees’ resilience and emotional management skills at an organizational level can reduce misunderstandings between supervisors and employees in their daily work. The negative effects of negative reciprocity are then reduced and defused, further weakening the negative effects of abusive supervision on employee voice behavior. For example, training courses on emotion regulation can be provided for employees to reduce their sensitivity and increase their stress tolerance. Reasonable ways and channels of communication can be established in the organization as an outlet for employees to express and regulate their emotions [52].

Finally, leaders should learn to use work engagement as a motivational mechanism to promote employees’ voice behavior. They should also learn to enhance organizational support to motivate employees, stimulate their autonomy at work [55], and address employees’ grievances and suggestions for organizational development at work in a timely manner. Replace abusive supervision with enlightened and supportive leadership to promote employees’ work engagement [10].

### 5.3. Limitations and Future Directions

This study has several limitations that future research is expected to address. First, although this study adopted a multistage approach to collecting data, homogeneity of data would still inevitably cause common method bias [72]. In the future, data could be collected from multiple sources. Specifically, employees evaluate the abusive supervision of leaders, and leaders evaluate the voice behavior of employees.

Second, abusive supervision is a subjective judgment made by employees, which may not be objective enough. This study focuses on the individual level, with individuals making evaluations and scores that may not be objective enough. In future research, we hope to explore the impact of abusive supervision on employee voice behavior by aggregating the evaluations of abusive supervision into a whole from the team level to improve the objectivity and reliability of the data.

Third, work engagement is a complex concept with three dimensions. This study only considers the concept as a whole and does not discuss it separately according to the three dimensions of vigor, dedication, and absorption. In fact, abusive supervision may have different effects on the three dimensions of work engagement. Future research should continue to discuss how abusive supervision specifically affects the three dimensions of work engagement, which in turn affect employee voice behavior.

Fourth, this study only explored the role of mediating variables between abusive supervision and the two types of employee voice behavior but did not address moderating variables. It has been suggested that the psychological climate plays a moderating role between abusive supervision and employee voice behavior [16]. It has also been suggested that employees’ perceived organizational support moderates the relationship between abusive supervision and voice behavior [54]. Therefore, future research could continue to explore under what boundary conditions the main effects in this study would be stronger or weaker.

Finally, due to the different cultural backgrounds between East and West, errors caused by cultural differences are inevitable in this study, so we expect that future studies will take this difference into account and conduct further studies on relevant topics.

## 6. Conclusions

Although there has been literature demonstrating the negative impact of abusive supervision on employee voice behavior, some studies have also concluded that the relationship between abusive supervision and voice behavior is in an inverted U-shape [71]. Due to the inconsistencies and research gaps of former studies, we empirically tested the model and concluded that (1) abusive supervision has a negative effect on both promotive and prohibitive voice behavior. Meanwhile, previous research usually took voice behavior as a whole and did not discuss the effect of abusive supervision on different dimensions of voice behavior. Therefore, this study attempts to discuss how abusive supervision affects promotive voice and prohibitive voice in different ways. Based on conservation of resources theory and social exchange theory, this study made hypotheses and obtained the following two conclusions: (2) work engagement partially mediates the relationship between abusive supervision and promotive voice behavior, indicating that when employees are subject to abusive supervision, they will reduce work engagement from the perspective of protecting their own psychological resources, thus reducing promotive voice behavior that contributes to the efficiency of the organizations, (3) while negative reciprocity partially mediates the negative relationship between abusive supervision and prohibitive voice behavior.

The findings revealed in this study deepen the theoretical understanding of employee voice behavior. The study also extends the research on abusive supervision to two dimensions of voice behavior by introducing work engagement and negative reciprocity as mediating variables. Finally, this study introduces work engagement as a mediator based on resource conservation theory and negative reciprocity as a mediator based on social exchange theory, expanding both theories to the study of abusive supervision on voice behavior.

## Figures and Tables

**Table 1:** Means, standard deviations, and interrelations of variables.

Variables	M	SD	1	2	3	4	5	6	7	8
1. Gender(T1)	0.52	0.50								
2. Age(T1)	40.44	9.47	−0.05							
3. Education(T1)	3.64	0.88	0.01	−0.20 **						
4. OT(T1)	4.37	2.17	−0.02	0.69 **	−0.13 **					
5. AS(T1)	2.09	0.73	−0.12 **	−0.10	−0.09	−0.09				
6. WE(T1)	3.61	0.80	0.01	−0.03	0.11 *	0.05	−0.22 **			
7. NR(T1)	3.07	0.81	−0.04	−0.15 **	−0.05	−0.21 **	0.15 **	−0.17 **		
8. PromV(T2)	3.78	0.83	0.09	0.12 *	0.07	0.14 **	−0.27 **	0.53 **	−0.12 **	
9.ProhV(T2)	3.60	0.80	0.09	0.03	0.05	0.07	−0.20 **	0.54 **	−0.15 **	0.86 **

Notes. N = 334. OT = organizational tenure; AS = abusive supervision; WE = work engagement; NR= negative reciprocity; PromV = promotive voice; ProhV = prohibitive voice. * *p* < 0.05; ** *p* < 0.01.

**Table 2:** Confirmatory factor analyses.

Factor Structure	χ^2^/df	RMSEA	CFI	TLI	SRMR
Five-factor model (abusive supervision; work engagement; negative reciprocity beliefs; promotive voice; prohibitive voice)	2.416	0.065	0.895	0.889	0.054
Four-factor model (combining promotive voice and prohibitive voice together)	2.514	0.067	0.887	0.881	0.055
Three-factor model (combining work engagement and negative reciprocity beliefs together)	5.382	0.115	0.673	0.655	0.174
Two-factor model (combining voice behavior, work engagement and negative reciprocity beliefs together)	7.572	0.140	0.508	0.483	0.178
Two-factor model (combining abusive supervision, work engagement and negative reciprocity beliefs together)	7.421	0.139	0.519	0.495	0.188
One-factor model (combining all items into one factor together)	9.292	0.158	0.379	0.348	0.201

Note. CFI, Comparative Fit Index; TLI, Tucker–Lewis Index; RMSEA, root-mean squared error of approximation; SRMR, standardized root mean square residual.

**Table 4:** Regression analysis of the mediating effect of work engagement.

Effect (AS → WE → PromV)	B	SE	LLCI	ULCI
Direct effect of X on Y	−0.16 **	0.05	−0.27	−0.06
Indirect effect of X on Y	−0.12 **	0.04	−0.20	−0.06
Total effect of X on Y	−0.28 **	0.06	−0.4	−0.16

** *p* < 0.01.

**Table 5:** Regression analysis of the mediating effect of negative reciprocity.

Effect (AS → NR → ProhV)	B	SE	LLCI	ULCI
Direct effect of X on Y	−0.19 **	0.06	−0.31	−0.07
Indirect effect of X on Y	−0.01 **	0.01	−0.05	−0.001
Total effect of X on Y	−0.20 **	0.06	−0.32	−0.08

** *p* < 0.01.

## Data Availability

The data presented in this study are available on request from the corresponding author. The data are not publicly available due to respondents’ privacy.
